# A Synergy-Based Optimally Designed Sensing Glove for Functional Grasp Recognition

**DOI:** 10.3390/s16060811

**Published:** 2016-06-02

**Authors:** Simone Ciotti, Edoardo Battaglia, Nicola Carbonaro, Antonio Bicchi, Alessandro Tognetti, Matteo Bianchi

**Affiliations:** 1 Centro di Ricerca “E. Piaggio”, University of Pisa, Largo Lucio Lazzarino 1, Pisa 56126, Italy; simone.ciotti@ing.unipi.it (S.C.); e.battaglia@centropiaggio.unipi.it (E.B.); nicola.carbonaro@centropiaggio.unipi.it (N.C.); bicchi@centropiaggio.unipi.it (A.B.); a.tognetti@centropiaggio.unipi.it (A.T.); 2 Advanced Robotics Department, Istituto Italiano di Tecnologia, via Morego 30, Genova 16163, Italy; 3 Information Engineering Department, University of Pisa, via G. Caruso 16, Pisa 56122, Italy

**Keywords:** kinematic wearable sensing, human hand synergies, under-sensing, optimal design

## Abstract

Achieving accurate and reliable kinematic hand pose reconstructions represents a challenging task. The main reason for this is the complexity of hand biomechanics, where several degrees of freedom are distributed along a continuous deformable structure. Wearable sensing can represent a viable solution to tackle this issue, since it enables a more natural kinematic monitoring. However, the intrinsic accuracy (as well as the number of sensing elements) of wearable hand pose reconstruction (HPR) systems can be severely limited by ergonomics and cost considerations. In this paper, we combined the theoretical foundations of the optimal design of HPR devices based on hand synergy information, *i.e.*, the inter-joint covariation patterns, with textile goniometers based on knitted piezoresistive fabrics (KPF) technology, to develop, for the first time, an optimally-designed under-sensed glove for measuring hand kinematics. We used only five sensors optimally placed on the hand and completed hand pose reconstruction (described according to a kinematic model with 19 degrees of freedom) leveraging upon synergistic information. The reconstructions we obtained from five different subjects were used to implement an unsupervised method for the recognition of eight functional grasps, showing a high degree of accuracy and robustness.

## 1. Introduction

The human hand is the primary tool to interact with the external world. Given its fundamental role in daily life activities, it is not surprising that much effort has been put into measuring its kinematics, with the two-fold goal of understanding the underpinning neuroscientific control mechanisms and to develop effective human-machine interfaces, with promising applications in rehabilitation, virtual reality, musical performance, video games, teleoperation and robotics [1,2].

However, accurate kinematic recordings through hand pose reconstruction systems (HPR) are difficult to achieve, due to the large number of degrees of freedom (DOFs) involved along a continuously-compliant structure. Roughly speaking, HPR systems common in the state of art can be divided into remote and wearable systems (see [1,3] for a survey). The former category comprises systems that are mostly visual based, while in the latter fall in glove-based systems. The design of HPR systems in general has to deal with a trade-off between accuracy and other factors, such as ergonomics and cost. While visual-based HPR systems are inexpensive, unobtrusive and typically very usable, their reconstruction capabilities are still far from an effective usage for continuous monitoring of daily life activities, since they are usually not mobile.

For this reason, in the following, we will focus on wearable systems, since they are the most suitable and commonly-employed tool in neuroscientific studies and rehabilitation. Indeed, a correct investigation of human mechanisms underpinning motor control of the human hand requires ecological instruments to analyze its kinematics, to open novel insights into the underlying neural control strategies [4]. At the same time, ambulatory and daily life monitoring of the residual sensorimotor hand function and its recovery is fundamental for optimal guidance and evaluation of the rehabilitation therapy. As highlighted in [5,6,7], one of the main requirements for hand monitoring in stroke patients, identified through questionnaires and interviews held with professionals and patients, is the quantification of the frequency and type of grasp of the affected and unaffected arms during daily activities. In the same studies, such a quantification was performed through continuous classification of a set of basic hand functional poses defined by Lister [8], which are related to the most common interaction tasks with the external environment. This type of information on grasp performance is not easy to obtain in daily life monitoring with current systems, which are often cumbersome and obtrusive.

To tackle this issue, in our recent works [9,10], we focused on the development of unobtrusive and wearable interfaces based on textile goniometers (knitted piezoresistive fabrics (KPF) technology) for monitoring human hand activity in daily life. In [11], we presented a glove specifically designed for daily life assessment of post-stroke recovery. In this system, KPF goniometers covered the metacarpal-phalangeal joints of the index and middle fingers and the trapezio-metacarpal joint of the thumb, exhibiting reliable performance in angular measurements.

However, despite these encouraging outcomes, the KPF goniometer technology still presents a few disadvantages, which are principally due to the high number of electrical connections per DOF, thus limiting the usage of such technological solutions for multi-DOF recordings. Considering these limitations (*i.e.*, a limited number of sensors can be integrated), the KPF glove was not suitable for a complete reconstruction of hand kinematics, but only for hand posture recognition from a discrete set of functional poses [7].

To improve HPR performance, an important asset is the theoretical work laid out in [12,13], where the existence of postural synergies was exploited to enhance kinematic and joint angle reconstruction performance and to design optimized gloves with a limited number of sensing elements, respectively. The basic idea was to interpret postural synergies, *i.e.*, goal-directed kinematic activation or inter-joint covariation patterns [4], in terms of statistical *a priori* information on the probabilistic distribution of human poses in common tasks, e.g., grasping. This information can be fused with insufficient and inaccurate measurements provided by an HPR to increase its performance [12] and can be used to design optimal placement of sensors on a glove for HPR in order to reconstruct hand posture, especially with a limited number of sensors [13]. However, these optimal design techniques were validated only in simulation. In [14], a hand avatar with 20 DOFs used in virtual grasping applications with two three-DOF haptic devices was animated only relying on the knowledge of kinematic constraints defined by hand postural synergies, but no sensor glove or advanced tracker with markers was used. To summarize, to the best of our knowledge, the techniques described in [12,13] represent the first attempt to use synergy information to advance reconstruction accuracy, *i.e.*, the estimation of the values of the whole joint angle set, using a limited number of measurements provided by a kinematic sensing system HPR.

In our preliminary work [15], we applied the synergy-based estimation technique to the measurements provided by the above-described KPF sensing glove. The goal was to reconstruct the hand kinematics according to a 15-DOF model, using postural synergies to complete information from only three measurements. The results of grasp reconstruction were encouraging, although preliminary, since we performed only a qualitative assessment on the reconstructed poses of one subject.

In this work, we pushed forward our investigation and designed and engineered, for the first time, a sensing glove based on five KPF goniometers, measuring five joint angles, whose placement on the human hand was inspired by the optimal design guidelines described in the theoretical work in [13]. The KPF goniometers were specifically built and integrated into the glove to fulfill the design requirements (*i.e.*, sensor length). The measurements they provided were completed through synergy-based estimation techniques to obtain hand posture reconstruction according to a 19-DOF model. The results of the reconstruction were visually represented through a 3D rendering, showing a very good agreement with real hand poses. In addition, the reconstructions we obtained from five different subjects were used to implement an unsupervised method for the recognition of eight functional grasps, showing a high degree of accuracy and robustness.

For the sake of clarity, let us stress two points. First, we would like to underline again that, although here, we applied the synergy-based HPR method for the reconstruction of eight poses typically used for sensorimotor functionality evaluation, it can be applied to any type of grasp, since it relies on *a priori* synergistic information observed in grasping tasks (see also the Discussion and Conclusions). The effectiveness of this method was demonstrated in [12], where the interested reader can also find a thorough comparison and discussion of the HPR state of the art. Second, to make the novelty of this paper clearer, we would like to emphasize again that, although it capitalizes on our previous results, this work provides an important original technological contribution. Indeed, in [12,16], the synergy-based pose reconstruction techniques were applied to an existing glove with a different sensing technology (non-optimal glove based on conductive elastomer sensors). The effectiveness of the optimal design techniques in [13,17] was demonstrated only through simulations. Finally, in the very preliminary work presented in [15], we applied the synergy-based hand pose reconstruction techniques to a glove equipped with three KPF sensors placed in a non-optimal fashion over the user’s hand, and the results we reported there were only qualitative. In this paper, we combine the theoretical foundations of synergy-inspired HPR discussed in [12,13,16,17] of synergy-inspired HPR and KPF sensors to realize a physical device for hand pose sensing, whose effectiveness we demonstrate in a quantitative manner with human experiments.

## 2. Materials and Methods

### 2.1. Theoretical Background

In this sub-section, we describe the theoretical aspects of hand pose reconstruction and optimal HPR systems design based on hand postural synergy.

#### 2.1.1. Synergy-Based Hand Pose Reconstruction

For the sake of clarity, let us summarize the definitions and results from [16]. Let us consider an *n* degrees of freedom kinematic hand model, with $y\in R^{m}$ measures provided by an HPR system. In this case, the joint variables $x\in R^{n}$ and measurements *y* are related by the equation:

(1) y=Hx+ν

with $H\in R^{m\times n}$ (m<n) the full row rank matrix and $v\in R^{m}$ the vector of measurement noise, with a zero mean and Gaussian distribution with covariance matrix *R*.

Our objective is to determine hand posture, which can be represented by the joint angles *x*, from a reduced set of measures *y*. This objective can be achieved by using postural synergy information. Hand synergies are goal-directed, combining muscle and kinematic activation, leading to a reduction of the dimensionality of the motor and sensory space [18]. In robotics, hand synergies have represented a highly effective solution for the fast and simplified design and control of artificial systems [19,20].

Under a kinematic point of view, hand synergies can be defined in terms of inter-joint covariation patterns, which were observed both in free hand motion [4] and object manipulation [21]. In [12], following the approach introduced in [4], we embedded synergy information in an *a priori* grasp set that we obtained using the PhaseSpace motion capture system (PhaseSpace Inc., San Leandro, CA, USA). In particular, we asked experiment participants to grasp a set of imagined objects and collected a large number *N* of postures, into a matrix $X\in R^{n\times N}$.

This information can be summarized in a covariance matrix $P_{o}\in R^{n\times n}$, which is a symmetric matrix computed as $P_{o}=\frac{\left(X-\bar{x}\right){\left(X-\bar{x}\right)}^{T}}{N-1}$, where $\bar{x}$ is a matrix n×N whose columns contain the mean values for each joint angle arranged in vector $\mu_{o}\in R^{n}$.

According to [16], the hand pose reconstruction can be obtained through the minimum variance estimation (MVE) technique as:

(2) $$\hat{x}={\left(P_{o}^{-1}+H^{T}R^{-1}H\right)}^{-1}\left(H^{T}R^{-1}y+P_{o}^{-1}\mu_{o}\right)\;$$

where matrix $P_{p}={\left(P_{o}^{-1}+H^{T}R^{-1}H\right)}^{-1}$ is the *a posteriori* covariance matrix. Equation (2) can be rewritten as:

(3) $$\hat{x}=\mu_{o}-P_{o}H^{T}{\left(HP_{o}H^{T}+R\right)}^{-1}\left(H\mu_{o}-y\right)\;$$

and the *a posteriori* covariance matrix becomes $P_{p}=P_{o}-P_{o}H^{T}{\left(HP_{o}H^{T}+R\right)}^{-1}HP_{o}$.

#### 2.1.2. Synergy-Based Optimal HPR System Design

The *a posteriori* covariance matrix depends on the measurement matrix *H* and can be used as a measure of how much information an observable variable carries about unknown parameters. In [13,17], we explored the role of the measurement matrix *H* on the estimation procedure and obtained as a result the optimal placement of sensors for a sensing device in order to obtain the maximum amount of the information on the complete hand posture.

In the ideal case of measures with no noise (R=0), the covariance matrix $P_{p}$ becomes a zero matrix when *H* is full rank, which means that the measures contain complete information on the hand posture. However, in the case of noisy measures and/or when the number of measurements *m* is less than the hand model DOFs *n*, the covariance matrix is not null. In these cases, we can consider the problem of finding the optimal matrix $H^{*}$ such that the hand posture information contained in it is maximized. Without loss of generality, we assume *H* to be full row rank and consider the problem of finding $H\in R^{m\times n}$, with m<n, which minimizes the squared Frobenius norm of $P_{p}$.

### 2.2. Sensors: KPF Goniometers

**Figure 1:**
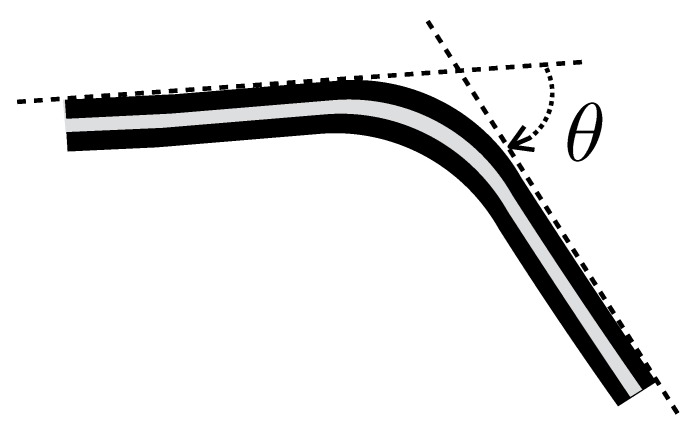
Representation of a double-layer textile goniometer. The two external stripes represent the piezoresistive layers. The grey line is the electrically-insulating layer. The bending angle (*θ*), represented by the angle between the tangent planes to the goniometer extremities (black dashed stripe), is proportional to the difference of the resistance (ΔR) of the sensing layers.

This section describes the working principle of the KPF goniometers that were optimally placed on the sensing glove. As reported in our previous research [9,11,15,22] and shown in Figure 1, textile goniometers consist of two piezoresistive layers connected through an electrically-insulating layer. The sensing layers of the textile goniometers were fabricated using knitted piezoresistive fabrics (KPFs), in previous work used as single-layer strain sensors for the extraction of biomechanical and cardio-pulmonary parameters [23,24].

As demonstrated in [11], the sensor output, the electrical resistance difference (ΔR) calculated from the two sensitive layers, is proportional to the flexion angle (*θ*), represented as the angle delimited by the tangent planes to the sensor extremities (Figure 1):

(4) ΔR=kθ

Equation (4) is verified if the two sensing layers are completely identical (geometrically and electrically equivalent). In practice, Equation (4) is not verified because the two sensing layers do not have the same electrical properties. In [11], the ΔR *vs*. *θ* relation was approximated by the following linear function:

(5) $$\Delta R=s_{\theta }\;\theta +\Delta R_{o}$$

where $s_{\theta }$ is the goniometer sensitivity, while $\Delta R_{0}$ is the offset. The angle *θ* can be obtained by the Equation (5) as:

(6) $$\theta =\frac{\Delta R-\Delta R_{o}}{s_{\theta }}=\alpha_{1}\;\Delta R+\alpha_{2}$$

The parameters $\alpha_{1}$ and $\alpha_{2}$, defined in Equation (6), are unknown and can be determined trough an initial calibration. The calibration phase consisted of acquiring the sensor output ($\Delta R_{1}$, $\Delta R_{2}$) in two known angular positions ($\theta_{1}$, $\theta_{2}$) to obtain $\alpha_{1}$ and $\alpha_{2}$.

### 2.3. The Sensing Glove

**Figure 2:**
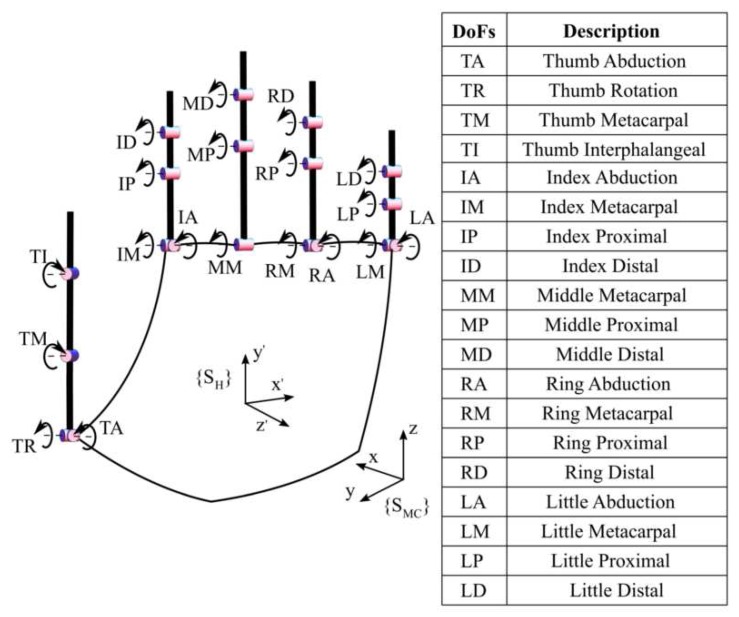
Kinematic model of the hand with 19 DOFs.

We built the sensing glove following the synergy-based optimal design guidelines reported in Section 2.1.2. In particular, we based the design on a 19-DOF kinematic model (n=19), inspired by the one described in [4,16] and reported in Figure 2.

It is worth noting that the original hand model in [4] had 15 DOFs and did not have the distal joints for the fingers. We completed the values of distal (D) joints, which are crucial to correctly visualize the shape of the hand posture during grasping, from proximal (P) joints, using the relationship $\theta_{D}=\frac{2}{3}\cdot \theta_{P}$ [25], thus obtaining a model with 19 DOFs.

We considered five measurements (m=5) obtained from five KPF goniometers. We chose to use five sensors as a good trade-off between the amount of information they enable to take from $P_{p}$ and the simplicity/wearability of the prototype. We placed each KPF goniometer so that each each measure $y_{j}$, j=1,⋯,m would correspond to a hand DOF, *i.e.*, a joint angle $x_{i}$, i=1,⋯,n. Note that this led to a full row rank measurement matrix (*H*), where each row is a vector of the canonical basis, with all entries except one equal to zero. In addition, we considered a negligible measurement noise (R=0; more specifically, acquiring 60 s of signals from the sensors and subtracting the average value, we obtained a standard deviation of less than 0.8deg).

**Figure 3:**
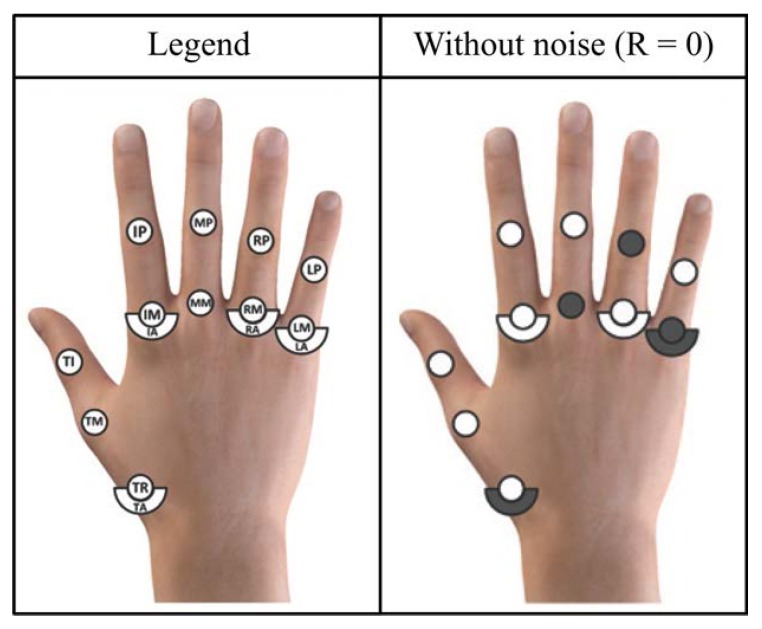
Measured joints (highlighted in color on the right) for the optimal design of a glove with five sensors, with R=0 (*cf.* Figure 2). These joints were selected from the results obtained in [13].

Here, the design problem was to find the optimal choice of the *m* DOFs to be measured (m=5). We solved the problem for the case of R=0, following the solution discussed in [13], to which the interested reader is referred for more details. Figure 3 reports the obtained optimal choice of DOFs to be measured, which includes the joints TA, MM, RP, LA and LM (see Figure 2).

**Figure 4:**
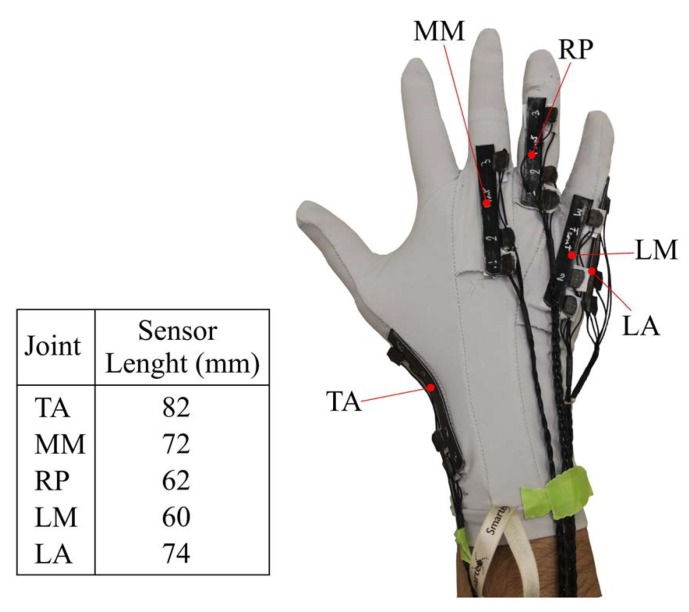
Glove with knitted piezoresistive fabrics (KPF) sensors on the joints of interest.

According to this optimal design, we built the five dedicated KPF goniometers and sewed the sensors on the glove, directly on the joints of interest. Geometric properties on the hand of experiment participants were considered to enable the correct measurement of the desired DOF, with particular care on the active length of each of the goniometers, which have to cover the corresponding joint without being influenced by the adjacent ones (to avoid cross talk). Possible artifacts due to sliding were minimized by the tight and elastic fabric of the glove. Figure 4 shows the sensing glove and provides information on the length of each sensor.

### 2.4. Data Acquisition

**Figure 5:**
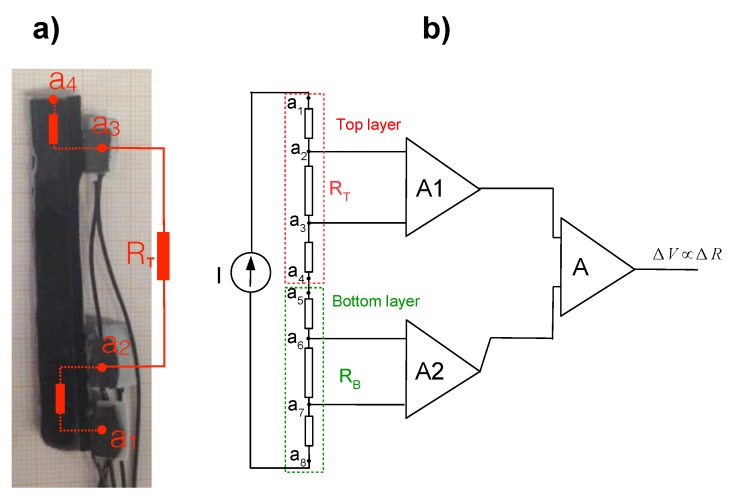
(**a**) A picture of the KPF goniometer showing the four pads for wiring connection; (**b**) diagram of the KPF goniometer acquisition electronics.

For the acquisition of ΔR from each of the five goniometers, we designed an *ad hoc* five-channel circuit. Each sensing layer was designed with four pads for sensor wiring ($a_{1}$ to $a_{4}$ for the top layer, $a_{5}$ to $a_{8}$ for the bottom layer, using a four-point resistance measurement method for the reduction of connection impedance) and can be represented as a series of three electrical resistances. Figure 5a shows the top sensing layer with its pads $a_{1}$ to $a_{4}$, while the complete diagram of the acquisition circuit is reported in Figure 5b. For each goniometer, the voltages $V_{T}=V_{a_{2}}-V_{a_{3}}$ and $V_{B}=V_{a_{6}}-V_{a_{7}}$ are measured between the internal pads when a constant and known current *I* is supplied through the external pads. A high input impedance stage, consisting of two instrumentation amplifiers (A1 and A2), measures the voltages across the piezoresistive sensors.

These voltages are proportional, through the known current *I*, to the resistances of the top and bottom layers ($R_{T}$ and $R_{B}$). A third differential amplifier *A* amplifies the difference between the measured voltages, obtaining the final output ΔV, which is proportional to ΔR and, thus, to the flexion angle *θ*. Each channel of the described front-end was analogically low pass filtered (anti-aliasing, cut-off frequency of 10 Hz). The resulting data were digitally converted (sample time of 100 Sa/s) and stored in a PC for off-line elaboration through a multichannel acquisition board (National Instrument, PCI 6071).

### 2.5. Experiments and K-Means Algorithm

Five right-handed (3 males, 2 females, age 25.8±1.10) volunteers took part in the experimental tests. All participants in these studies gave informed consent to perform the experiments. No subjects reported physical limitations that would affect their ability to perform the task. All data collected in this study were approved by the local ethical committee. The dimensions of their hands were in the same range (length 175±7.9 mm from the middle finger tip to the wrist, 205±26.9 mm width from the tip of the thumb to the tip of the little finger, in open hand posture). This allowed us to reduce the inaccuracies derived from sensor placement.

**Figure 6:**
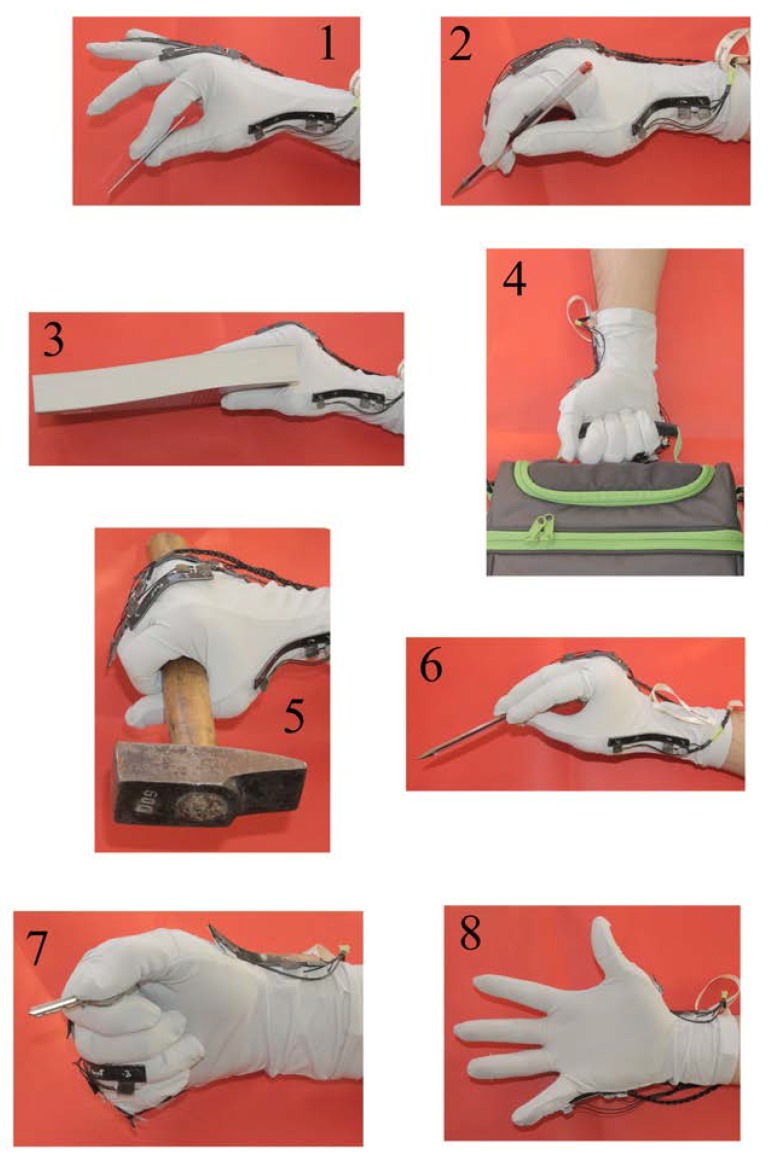
Types of grasp performed during the experiments.

Before starting the experiments, we calibrated the KPF goniometers (to find the $\alpha_{i}$ coefficients defined in Equation (6)) in two angular positions: a zero position, with the glove flat on a table, and a 90 degree position, with the sensors bent on a goniometer. Subjects were then instructed to perform the eight poses reported in Figure 6, which we chose among the ones described in [8]. We chose these poses, since they express in a quite exhaustive form the range of possible grasping actions in the activities of daily living [7,15]. At the beginning of each test, subjects were comfortably seated, with their forearm and hand on the table, palm down. An image of the target pose (as in Figure 6) was provided to the subjects, who performed the grasp as if the object was physically present. A visual reconstruction of the hand posture was provided to the subjects during each test.

**Figure 7:**
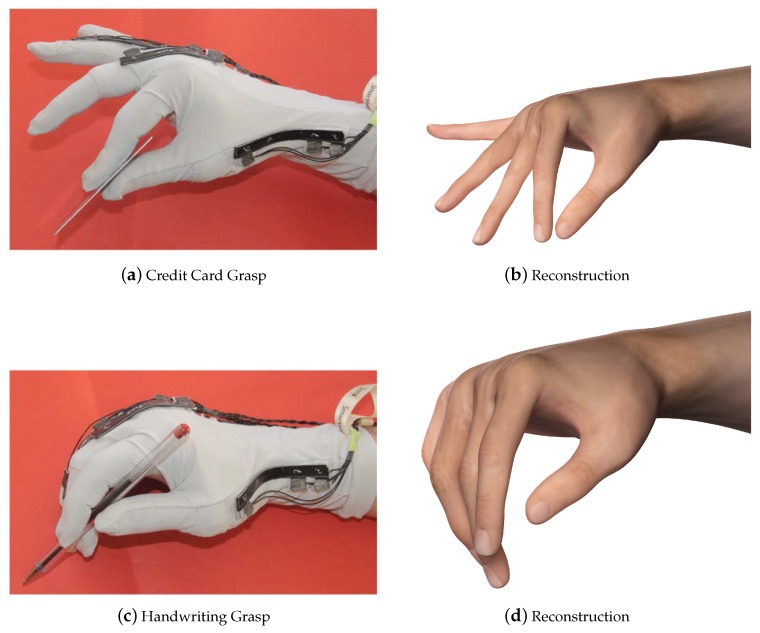

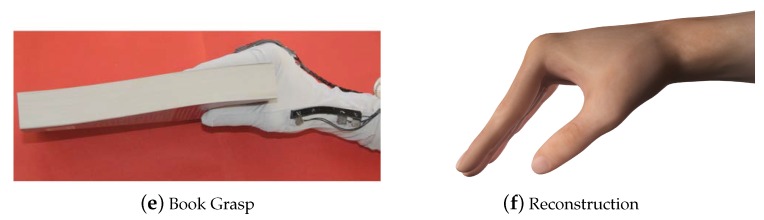
Comparison between real and reconstructed grasps (obtained from the five measurements extended to the 19-DOF pose and rendered in 3D Studio Max).

**Figure 8:**
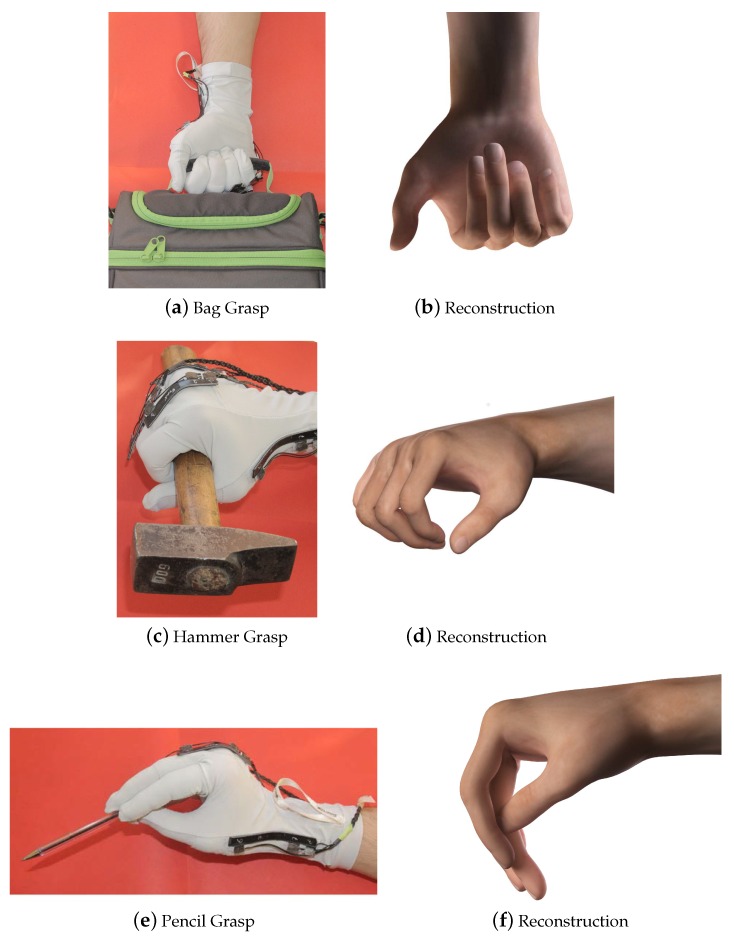
Comparison between real and reconstructed grasps (obtained from the five measurements extended to the 19-DOF pose and rendered in 3D Studio Max).

**Figure 9:**
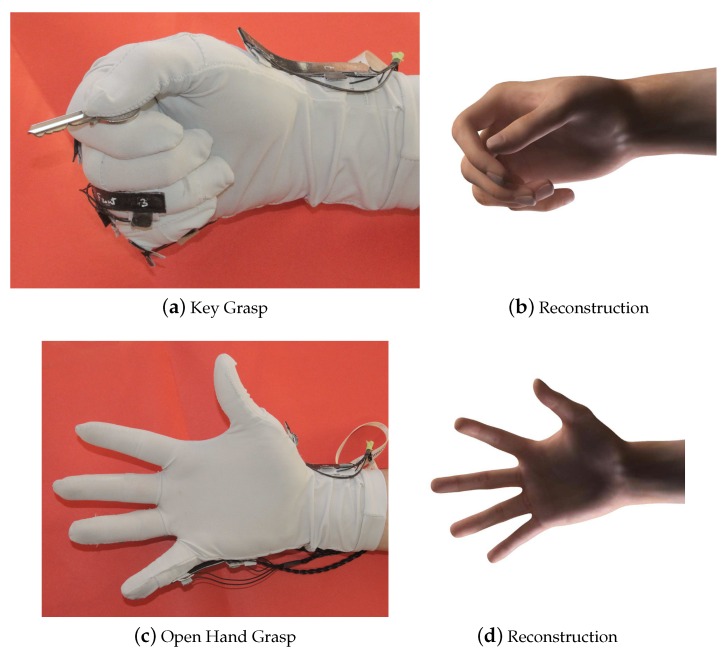
Comparison between real and reconstructed grasps (obtained from the five measurements extended to the 19-DOF pose and rendered in 3D Studio Max).

The order of the target poses was randomized, and each test was repeated three times by each participant, obtaining a total of 120 tests (*i.e.*, each grasp type was repeated 15 times). Each pose was held for 10 s, and we computed the mean value of the glove measurement vector over this time interval ($\bar{y}$). The hand kinematic reconstruction (*i.e.*, the vector *x* containing the 19 DOFs) was obtained completing the measurements of the glove ($\bar{y}$) with the procedure described in Section 2.1.1 (see Equation (3)). Sample results of the reconstructions are reported in Figure 7, Figure 8 and Figure 9.

The kinematic reconstructions obtained in this phase were then used in a *K-means* [26,27] single partition unsupervised algorithm. We chose the *K-means* algorithm since it is the simplest and most commonly-used algorithm employing a squared error criterion [26]. The *K-means* clustering algorithm performs the following operations:

1. Choose *k* (one for each target pose, *i.e.*, 8, in our case) cluster centroids starting locations;
2. Assign each sample to the closest cluster centroid;
3. Recompute the cluster centroid using the current cluster memberships;
4. If the convergence criterion is not satisfied, go to Step 2.

We chose to minimize the *squared Euclidean* distance measurement, *i.e.*, $d\left(x,c\right)=\left(x-c\right){\left(x-c\right)}^{\prime }$, where *x* is the sample and *c* the cluster centroid location. To validate recognition results, we used *cross-validation* [28]. In our work, we use a *leave-one-out* cross-validation (LOOCV) method: we applied the *K-means* algorithm a number of times equal to the number of participants, each time selecting the cluster centroid *c* as the average over three repetitions of the reconstructed poses from a volunteer, for a given grasp, and using data from the remaining volunteers as samples for the clustering.

## 3. Results

Table 1, Table 2, Table 3, Table 4 and Table 5 show the results of clustering performed with the techniques described in the previous section. In each table, each target pose is associated with one of the resulting clusters. The table Results: Volunteer *i* refers to the results obtained when data from the volunteer number *i* were used as starting locations for the cluster centroids, while data from the remaining volunteers were used as samples for the clustering process. The accuracy was evaluated for each pose (relative accuracy) and on the whole set (absolute). We considered as 100% accuracy when all of the grasps of each type are associated with the cluster generated from the corresponding centroid.

As the tables show, a different choice for the cluster centroid starting location can affect the accuracy of the classification results (e.g., see Table 1 and Table 3), since *K-means* outcomes tend to a local optimum [26]. For this reason, a correct choice of the cluster centroid start position is fundamental to obtain a good cluster identification and the correct grasp data-cluster association. This aspect is strictly connected on the operation performed by the *K-means* algorithm (see Section 2.5).

In Figure 7, Figure 8 and Figure 9, the comparisons between the experimental grasps, performed by a sample participant wearing the glove, and the rendered reconstructions of these grasps are reported. Note that the reconstructions are defined in $R^{19}$, completing the five measurements data with synergistic information. We can observe a high degree of similarity between the real grasps and the reconstructions. For the sake of visualization, pictures also show the object associated with each grasp.

Results exhibit an accuracy close to 100%, showing a high degree of robustness and generalization across different subjects. Slightly inferior results can be observed for Grasp Pose 2, which can be explained by the similarity w.r.t. Grasp Pose 6. This result is more evident for Subjects 3 and 4, whose hand dimensions are further away from the average dimensions of the participants, due to a non-ideal placement of the sensors on the joints to be measured or sensor sliding. This issue is discussed in the Discussion and Conclusions. The very positive results demonstrate that the optimal design and estimation, based on synergy information, led to a reliable under-sensed glove and hand pose reconstruction. It is important to note that we used only five sensors and measurements, while reconstruction and grasp type recognition were performed according to a 19-DOF kinematic model.

## 4. Discussion and Conclusions

This paper presents a sensing glove based on KPF sensors. In front of only five measurements, we have shown a good performance reconstruction of eight functional grasps, which led to an almost 100% accuracy of the unsupervised method that we implemented, w.r.t. a 19-DOF kinematic model, with a high degree of generalization across subjects. Furthermore, 3D reconstructions show a high degree of visual similarity to the real grasps. To obtain this goal, we leveraged upon optimal design and HPR enhancement techniques, which exploited the concept of synergies, *i.e.*, the fact that human hands, although very complex and possibly different, share many commonalities in how they are shaped and used in frequent everyday tasks.

The here presented approach was proven to successfully combine the reliability and wearability of KPF technologies, together with under-sensing strategies. In this manner, we could pave the path towards a novel paradigm of sensing systems that can adapt to deformable bodies (e.g., human and soft robots), with a limited number of sensing elements, leading to both a more ecological way of studying human kinematics and monitoring the state of soft manipulators. Ideal application fields of these outcomes can be neuroscientific investigation, therapeutic assessment and human robot interaction. In the literature, it is possible to find other examples of sensing gloves, which can be also applied to hand function assessment in daily life conditions.

The most common existing devices employ off-the-shelf flex sensors [29,30,31,32]. These prototypes use a limited number of flex sensors (e.g., four in [30] or ten in [31]), and the validation experiments usually focus on single sensor performance, without taking into account the whole hand pose reconstruction. On the contrary, our approach, despite the low number of sensors in use (*i.e.*, five), is able to completely reconstruct in a Bayesian optimal way hand kinematics (*i.e.*, according to the 19-DOF model reported in Figure 2). In addition, our KPF goniometers are stretchable, while other flex-sensors (usually) are not; hence, they cannot fully adapt to the compliant and deformable nature of the human hand.

The need for stretchability has pushed other works to adopt (single-layer) smart textiles [33] or to consider manufacturing techniques to print piezoresistive sensors directly on gloves [34,35]. However, as previously mentioned, to the best of our knowledge, there is no evidence about complete HPR. Moreover, this kind of sensor requires a more complex calibration phase to provide a direct measurement of joint angles. Other approaches for sensing gloves rely on the widespread diffusion of MEMS-based inertial measurement units [36,37,38]. The work in [36] is particularly relevant: it combines inertial measurement units with a hand kinematic model embedded in a Kalman-based reconstruction algorithm. The inertial-based approach is very promising; however, the performance strongly depends on the sensor-to-segment alignment, and the final design can be more obtrusive than our approach.

Of course, there are some intrinsic limitations of our approach. First, in order to improve reconstruction performance, we should take into account differences in user hand dimensions: gloves with different sensor lengths and dimensions are envisioned to tackle this issue. Second, to apply this method to different tasks other than grasp, we would need to leverage different *a priori* information: indeed, despite the many commonalities that human hands share, some tasks (e.g., playing piano or haptic exploration [39]) involve different actuation patterns that should be considered for a correct glove design. However, it is important to underline that the here presented synergy-inspired methods leveraging human grasp *a priori* information can be generalized to any type of grasp [12].

Future work will also aim to investigate these sensing strategies for hand rehabilitation purposes, such as therapy design and assessment. Under this regard, it is important to note that neurological disorders (such as stroke or focal dystonia) affect the ability to coordinate multi-joint movements, thus resulting in an abnormal coupling of body DOFs and in the use of a different or smaller number of physiological synergies [40]. These *pathological synergies* define different covariation schemes across hand joint angles w.r.t. physiological ones. For these reasons, our method could not be applied as it is, since the hand pose reconstruction we propose leverages *a priori* synergistic patterns identified in normal subjects and because of patient variability, but it can still be very useful for diagnostic procedures. Indeed, combining the here proposed techniques with other HPR methods, e.g., a complete sensing of hand joint angles, we could have both an ideal kinematic reconstruction of hand posture, *i.e.*, as it would be in physiological conditions and described in this work, and the actual/real pathological pose. In this manner, we could provide an effective benchmarking to evaluate the outcomes of the rehabilitative procedure and drive the design of more effective rehabilitation protocols.

Other applications of our results can be in robotic teleoperation [1,2] and in the entertainment field, such as video-games and virtual reality, where economic considerations are particularly strict and limit the number of sensors to use, in order to enable mass-diffusion devices. In this regard, it is worth noting that the techniques for optimal glove design described in the theoretical work in [13] and presented in the conference version in [17] were awarded with the JTCFnovel technology paper award for the amusement culture at the 2012 IEEE IROSconference. These techniques have been implemented for the first time in this paper, opening promising scenarios for further developments.

## Figures and Tables

**Table 1:** Results: Volunteer 1.

		Recognized Grasp								Relative Accuracy	Absolute Accuracy
		1	2	3	4	5	6	7	8		
Grasp Type	1	**12**	0	0	0	0	0	0	0	100%	100%
	2	0	**12**	0	0	0	0	0	0	100%	
	3	0	0	**12**	0	0	0	0	0	100%	
	4	0	0	0	**12**	0	0	0	0	100%	
	5	0	0	0	0	**12**	0	0	0	100%	
	6	0	0	0	0	0	**12**	0	0	100%	
	7	0	0	0	0	0	0	**12**	0	100%	
	8	0	0	0	0	0	0	0	**12**	100%	

**Table 2:** Results: Volunteer 2.

		Recognized Grasp								Relative Accuracy	Absolute Accuracy
		1	2	3	4	5	6	7	8		
Grasp Type	1	**12**	0	0	0	0	0	0	0	100%	100%
	2	0	**12**	0	0	0	0	0	0	100%	
	3	0	0	**12**	0	0	0	0	0	100%	
	4	0	0	0	**12**	0	0	0	0	100%	
	5	0	0	0	0	**12**	0	0	0	100%	
	6	0	0	0	0	0	**12**	0	0	100%	
	7	0	0	0	0	0	0	**12**	0	100%	
	8	0	0	0	0	0	0	0	**12**	100%	

**Table 3:** Results: Volunteer 3.

		Recognized Grasp								Relative Accuracy	Absolute Accuracy
		1	2	3	4	5	6	7	8		
Grasp Type	1	**12**	0	0	0	0	0	0	0	100%	95.83%
	2	0	**10**	0	0	0	**2**	0	0	83.33%	
	3	**1**	0	**11**	0	0	0	0	0	91.67%	
	4	0	0	0	**12**	0	0	0	0	100%	
	5	0	0	0	0	**12**	0	0	0	100%	
	6	0	0	0	**1**	0	**11**	0	0	91.67%	
	7	0	0	0	0	0	0	**12**	0	100%	
	8	0	0	0	0	0	0	0	**12**	100%	

**Table 4:** Results: Volunteer 4.

		Recognized Grasp								Relative Accuracy	Absolute Accuracy
		1	2	3	4	5	6	7	8		
Grasp Type	1	**12**	0	0	0	0	0	0	0	100%	95.83%
	2	0	**9**	0	0	0	**3**	0	0	75%	
	3	0	0	**12**	0	0	0	0	0	100%	
	4	0	0	0	**12**	0	0	0	0	100%	
	5	0	0	0	0	**12**	0	0	0	100%	
	6	0	0	0	**1**	0	**11**	0	0	91.67%	
	7	0	0	0	0	0	0	**12**	0	100%	
	8	0	0	0	0	0	0	0	**12**	100%	

**Table 5:** Results: Volunteer 5.

		Recognized Grasp								Relative Accuracy	Absolute Accuracy
		1	2	3	4	5	6	7	8		
Grasp Type	1	**12**	0	0	0	0	0	0	0	100%	98.96%
	2	0	**11**	0	0	0	**1**	0	0	91.67%	
	3	0	0	**12**	0	0	0	0	0	100%	
	4	0	0	0	**12**	0	0	0	0	100%	
	5	0	0	0	0	**12**	0	0	0	100%	
	6	0	0	0	0	0	**12**	0	0	100%	
	7	0	0	0	0	0	0	**12**	0	100%	
	8	0	0	0	0	0	0	0	**12**	100%	
